# TGF-beta signaling proteins and the Protein Ontology

**DOI:** 10.1186/1471-2105-10-S5-S3

**Published:** 2009-05-06

**Authors:** Cecilia N Arighi, Hongfang Liu, Darren A Natale, Winona C Barker, Harold Drabkin, Judith A Blake, Barry Smith, Cathy H Wu

**Affiliations:** 1 Delaware Biotechnology Institute, University of Delaware, Newark, DE, USA; 2 Georgetown University Medical Center, Washington DC, USA; 3 The Jackson Laboratory, Bar Harbor, ME, USA; 4 Center of Excellence in Bioinformatics and Life Sciences, and Department of Philosophy, University at Buffalo, NY, USA

## Abstract

**Background:**

The Protein Ontology (PRO) is designed as a formal and principled Open Biomedical Ontologies (OBO) Foundry ontology for proteins. The components of PRO extend from a classification of proteins on the basis of evolutionary relationships at the homeomorphic level to the representation of the multiple protein forms of a gene, including those resulting from alternative splicing, cleavage and/or post-translational modifications. Focusing specifically on the TGF-beta signaling proteins, we describe the building, curation, usage and dissemination of PRO.

**Results:**

PRO is manually curated on the basis of PrePRO, an automatically generated file with content derived from standard protein data sources. Manual curation ensures that the treatment of the protein classes and the internal and external relationships conform to the PRO framework. The current release of PRO is based upon experimental data from mouse and human proteins wherein equivalent protein forms are represented by single terms. In addition to the PRO ontology, the annotation of PRO terms is released as a separate PRO association file, which contains, for each given PRO term, an annotation from the experimentally characterized sub-types as well as the corresponding database identifiers and sequence coordinates. The annotations are added in the form of relationship to other ontologies. Whenever possible, equivalent forms in other species are listed to facilitate cross-species comparison. Splice and allelic variants, gene fusion products and modified protein forms are all represented as entities in the ontology. Therefore, PRO provides for the representation of protein entities and a resource for describing the associated data. This makes PRO useful both for proteomics studies where isoforms and modified forms must be differentiated, and for studies of biological pathways, where representations need to take account of the different ways in which the cascade of events may depend on specific protein modifications.

**Conclusion:**

PRO provides a framework for the formal representation of protein classes and protein forms in the OBO Foundry. It is designed to enable data retrieval and integration and machine reasoning at the molecular level of proteins, thereby facilitating cross-species comparisons, pathway analysis, disease modeling and the generation of new hypotheses.

## Background

Biomedical ontologies are emerging as critical tools in genomic and proteomic research, where complex data in disparate resources need to be integrated. The OBO Foundry is a collaborative effort to establish a set of principles for ontology development with the goal of creating a suite of orthogonal interoperable reference ontologies in the biomedical domain [1]. The Foundry ontologies are organized along two dimensions: (1) granularity (from molecule to population) and (2) relations to time (objects, qualities, processes). In this scheme, PRO is a representation of entities on the level of granularity of molecules, and interoperates with other ontologies, such as the Sequence Ontology (SO) [2] and the Gene Ontology (GO) [3] which incorporate representations of protein qualities and processes. PRO encompasses a sub-ontology of proteins based on evolutionary relatedness (ProEvo) and a sub-ontology of the multiple protein forms produced from a given gene (ProForm) [4]. Of two other ontologies that have been designed for protein database integration or annotation, neither includes representations of the protein forms themselves. The Protein Ontology (PO) [5] includes terms and relationships to describe attributes of individual protein forms (such as physicochemical properties), while the Proteomics Process Ontology (ProPreO) [6] serves the detailed description of proteomics experimental processes.

Here we summarize the current PRO framework and the accompanying annotations, basing our account of the use of the PRO in representating proteins from the TGF-beta signaling pathway. This pathway is well-studied, and thus provides a rich body of protein annotations relating to a wide spectrum of protein forms derived from cleavage and/or post-translational modifications (PTMs), alternative splicing, and sequence variants that are related to disease.

### The PRO framework

**Figure 1:**
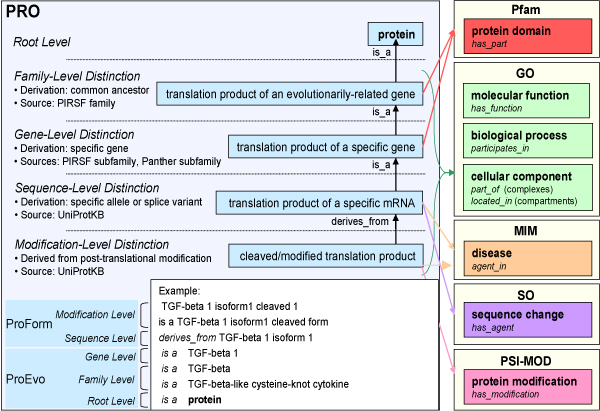
**PRO framework**. Current working model and a subset of the possible connections to other ontologies.

A detailed description of the PRO framework has been presented in our previous work [4]. Briefly, the framework was designed to enable data retrieval and integration, and machine reasoning at the molecular level of proteins by means of a structure to support formal, computer-based inferences of shared attributes among homologous proteins (addressed by ProEvo); and an explicit representation of the various forms of a given gene product (addressed by ProForm) [4]. Figure 1 is an outline representation of the current version of PRO and of a subset of its connections to other ontologies. The root in the ontology is the class *protein*, which is defined as "A biological macromolecule that is composed of amino acids linked in a linear sequence (a polypeptide chain), and is genetically encoded". PRO terms are connected by the relationship *is_a *or *derives_from*, both defined in the OBO Relations Ontology [7]. The framework figure points to levels of distinction that are "unofficial" descriptors for sets of PRO classes meant to provide some indication of how PRO is organized.

#### ProEvo

Proteins with full-length sequence similarity are said to be homeomorphic; they are presumed to share a common ancestor. Within any given homeomorphic group, there may be monophyletic subgroups of proteins that have distinct functions [8,9]. ProEvo was designed to define protein classes on this basis and to capture the relationship between these classes. Therefore, it includes proteins at both the family and the gene product levels. In ProEvo, terms are connected by the *is_a *relationship.

#### ProForm

This part of the ontology describes the translational products that are experimentally characterized, and includes definitions of sequence forms arising from allelic, splice and translational variation and from PTM and cleavage. It also includes representations of protein products of fused genes. Therefore, the coverage domain of ProForm includes both sequence (isoform and variant) and modification levels. We use the *derives_from *relationship to describe the relation between a modified form and the parent protein.

### PRO annotation

The generation of protein diversity from a single gene can make the systematic functional annotation of gene products difficult. Many model organism databases (MODs) utilize the Gene Ontology (GO) for functional annotation of gene products. Annotations provided in gene association files for each organism are tied to a single object: gene, transcript or protein. However, most MOD annotations currently default to the level of the gene. Similarly, most protein annotations in GOA are attached to the canonical sequence, and in a few cases to the specific isoform [10]. PRO defines existing protein objects based on the current knowledge, allowing annotation at a more appropriate level (such as isoforms, sequence variants and post-translationally modified forms). As depicted in Figure 1, the attributes of each PRO term can be described by cross-referencing to various ontologies and/or databases that are pertinent to protein annotation such as GO, SO and Modification Ontology (PSI-MOD).

## Results and discussion

### Building the ontology

Here we focus on the set of proteins in the TGF-beta signaling pathway as described in the KEGG pathway database [11], which consists of three sub-pathways: the TGF-beta signaling pathway, the bone morphogenetic protein signaling pathway, and the activin-mediated signaling pathway. It includes reference to 79 human/mouse orthologous proteins that map to 34 PIRSF [8] homeomorphic families and 36 Pfam [12] domains. We applied an automated process to generate the prospective PRO nodes, followed by manual curation involving literature review and further sequence analysis. Each curated node consists of a PRO ID, a term name, a definition and a cross-reference (when applicable). The framework allows the creation of ProForm nodes for newly published, experimentally-characterized isoforms or sequence variants, including those not yet represented in sequence databases (e.g., PRO:000000478 smad5 isoform 2 and PRO:000000483 smad9 isoform 2). Moreover, it allows the representation of proteins that are products of a gene fusion due to chromosomal translocation, such as PRO:000000091 *creb-binding protein/zinc finger protein HRX *that is encoded by part of the CREBBP gene at the N-terminus and part of the MYST4 gene at the C-terminus. This form is observed in some cases of acute myelogenous leukemia.

**Figure 2:**
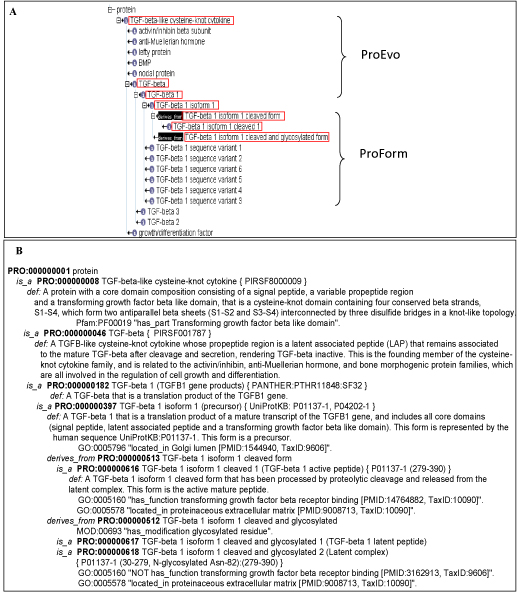
**Ontology for the TGF-beta 1 protein**. **A**. Snapshot of the ontology (partial DAG view) in OBO Edit 1.1 including terms representing ProEvo and ProForm. Terms in red boxes are described in panel B. **B**. Detailed example of the ontology and annotation displayed together for convenience. The above is a partial view, not all forms are listed, and only key annotations are shown.

Figure 2A shows a snapshot of the ontology in OBO edit with delineation of ProEvo and ProForm for the TGF-beta 1 protein. The important property for each ProForm term is simply that the protein form has been found to exist in nature, not how that form came into existence. Thus, PRO does not take into account the steps leading to a particular form, and there is no hierarchy indicating that, for example, a phosphorylated and ubiquitinated form derived from an original phosphorylated form–the hierarchy is flattened so that each term is a sibling of the other. This is because one cannot assume that the steps leading to a multiply-modified form always occur in the same order.

### PRO annotation

We have created a PRO association file (PAF) to provide the experimentally-based annotation for the proteins that are sub-types of a given class. To facilitate the exchange of annotation between PRO and GO, the PAF adopts the format of the GO annotation file [13] with some modifications. For example, each PRO term may contain annotation derived from other ontologies in addition to GO. Additional columns were added to account for sequence coordinate specifications, such as the range of the sequence (for cleaved form) or the modified residue(s) (for modified form) (Table 1).

**Table 1:** Example annotation in the PRO association file (PAF).

**PRO_ID**	**Object_term**	**Qual**	**Relation**	**Ontology_ID**	**Ontology_term**	**Ev.Source (PMID)**	**DB_ID (UniProt)**	**Modified_residue, MOD_ID**
**PRO:000000535**	c-myc isoform 1 acetylated 1		has_function	GO:0003700	transcription factor activity	16126174	P01106-1	Lys-143/Lys-157/Lys-275/Lys-317/Lys-323/Lys-371, MOD:00394
**PRO:000000535**	c-myc isoform 1 acetylated 1		located_in	GO:0005634	nucleus	16126174	P01106-1	Lys-143/Lys-157/Lys-275/Lys-317/Lys-323/Lys-371, MOD:00394
**PRO:000000535**	c-myc isoform 1 acetylated 1		part_of	GO:0005667	transcription factor complex	16126174	P01106-1	Lys-143/Lys-157/Lys-275/Lys-317/Lys-323/Lys-371, MOD:00394
**PRO:000000535**	c-myc isoform 1 acetylated 1		has_modification	MOD:00394	acetylated residue	16126174	P01106-1	Lys-143/Lys-157/Lys-275/Lys-317/Lys-323/Lys-371, MOD:00394
**PRO:000000536**	c-myc isoform 1 glycosylated 1		has_function	GO:0003700	transcription factor activity	11904304	P01106-1	Thr-58, MOD:00806
**PRO:000000536**	c-myc isoform 1 glycosylated 1		participates_in	GO:0006357	regulation of transcription from RNA polymerase II promoter	11904304	P01106-1	Thr-58, MOD:00806
**PRO:000000536**	c-myc isoform 1 glycosylated 1		has_modification	MOD:00806	O-(N-acetylaminoglucosyl)-L-threonine	11904304	P01106-1	Thr-58, MOD:00806
**PRO:000000537**	c-myc isoform 1 phosphorylated 1		has_function	GO:0003700	transcription factor activity	11018017	P01108-1	Ser-62, MOD:00046
**PRO:000000537**	c-myc isoform 1 phosphorylated 1		located_in	GO:0005634	nucleus	11018017	P01108-1	Ser-62, MOD:00046
**PRO:000000537**	c-myc isoform 1 phosphorylated 1		part_of	GO:0005667	transcription factor complex	11018017	P01108-1	Ser-62, MOD:00046
**PRO:000000537**	c-myc isoform 1 phosphorylated 1		participates_in	GO:0006357	regulation of transcription from RNA polymerase II promoter	11018017	P01108-1	Ser-62, MOD:00046
**PRO:000000537**	c-myc isoform 1 phosphorylated 1		participates_in	GO:0008283	cell proliferation	11018017	P01108-1	Ser-62, MOD:00046
**PRO:000000537**	c-myc isoform 1 phosphorylated 1		participates_in	GO:0055072	iron ion homeostasis	11018017	P01108-1	Ser-62, MOD:00046
**PRO:000000537**	c-myc isoform 1 phosphorylated 1		has_modification	MOD:00046	O-phospho-L-serine	11018017	P01108-1	Ser-62, MOD:00046
**PRO:000000538**	c-myc isoform 1 phosphorylated 2	NOT	has_function	GO:0003700	transcription factor activity	7623799	P01106-1	Ser-62, MOD:00046|Thr-58, MOD:00047
**PRO:000000538**	c-myc isoform 1 phosphorylated 2		located_in	GO:0005634	nucleus	14563837	P01108-1	Ser-62, MOD:00046|Thr-58, MOD:00047
**PRO:000000538**	c-myc isoform 1 phosphorylated 2		has_modification	MOD:00046	O-phospho-L-serine	14563837	P01108-1	Ser-62, MOD:00046|Thr-58, MOD:00047
**PRO:000000538**	c-myc isoform 1 phosphorylated 2		has_modification	MOD:00047	O-phospho-L-threonine	14563837	P01108-1	Ser-62, MOD:00046|Thr-58, MOD:00047
**PRO:000000539**	c-myc isoform 1 phosphorylated 3		located_in	GO:0005634	nucleus	12676581	P01106-1	Ser-62/Ser-71, MOD:00046
**PRO:000000539**	c-myc isoform 1 phosphorylated 3		participates_in	GO:0045766	positive regulation of angiogenesis	12676581	P01106-1	Ser-62/Ser-71, MOD:00046
**PRO:000000539**	c-myc isoform 1 phosphorylated 3		has_modification	MOD:00046	O-phospho-L-serine	12676581	P01106-1	Ser-62/Ser-71, MOD:00046
**PRO:000000480**	smad6 isoform 2	NOT	has_part	Pfam:PF03165	MH1 domain	11284962	O43541-2	

Selected columns are shown. Note that the last two columns are a tuple that define the sub-type of the class.

### PRO applications

The PRO framework provides a basis for more accurate annotation. This is especially important given the current growth of experimental data specific to the isoforms and modified forms. Figure 2 shows the PRO terms related to the TGF-beta 1 protein together with definitions, relationships and annotations, demonstrating the complexity and variety of protein classes and sequence forms that can be derived from a given parent sequence. At the ProEvo level, the PRO term *TGF-beta-like cysteine-knot cytokine *is defined as a protein with a signal peptide, a variable propeptide region and a cysteine-knot domain (Figure 2B, PRO:000000008). The class represented by this term has seven children nodes (Figure 2A), each defined as a separate group on the basis of sequence similarity and distinctive functional features. The granularity at this level varies depending on the sequence and functional diversity of the protein class. However, a ProEvo leaf node is always represented by a gene product class, which is defined as all protein products of orthologous genes. TGF-beta is a child node of the above-mentioned class and has three leaf nodes, TGF-beta 1, TGF-beta 2 and TGF-beta 3. In the current framework, PRO represents the full-length homeomorphic proteins and not the individual domains. Instead, domain information is included as a cross-reference to the Pfam domain database [12] in the ontology to indicate that a given protein class *has_part *some domain (Figure 2B, PRO:000000008). At the ProForm level, the TGF-beta 1 precursor (PRO:000000397) is a dimer and undergoes cleavages by a signal peptidase and by furin in the Golgi to generate two functionally important chains: the TGF-beta 1 mature peptide (PRO:000000616) and the latent peptide (PRO:000000617). These two chains remain associated as a latent complex (PRO:000000618) until proteases in the extracellular space degrade the latent peptide. In most databases, TGF-beta 1 canonical sequence (corresponding to the precursor) is annotated with GO:0005160 transforming growth factor beta receptor binding. ProForm allows this GO term to be appropriately associated with the active mature protein rather than the precursor. Note that these various forms not only differ in function but also in their cellular localization.

#### PRO and cross-species analysis

**Figure 3:**
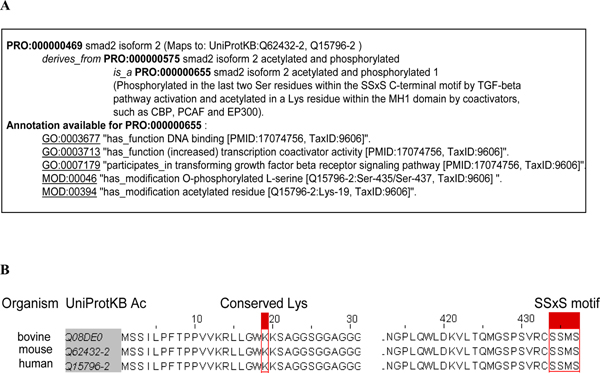
**PRO for cross-species comparison**. **A**. Ontology and annotation for smad2 isoform 2 protein. **B**. Multiple sequence alignment of the N- and C-termini of smad2 protein isoform 2 orthologs containing the modified sites.

PRO can facilitate cross-species comparison of protein forms based on annotations with experimental evidence and sequence conservation, as illustrated in Figure 3. Here, PRO:000000655 (smad2 isoform 2 acetylated and phosphorylated 1) is annotated with GO terms based on the experimental evidence on the human entry (with the associated literature and taxon ID) (Figure 3A). Based on the PRO mapping to UniProtKB, there is a mouse counterpart for smad2 isoform 2. Since the protein features (acetylation and phosphorylation sites) are conserved, (Figure 3B), one can investigate the existence of this modified form in mouse and its possible regulation by the coactivators. This analysis is not limited to human and mouse, since a sequence search using the human isoform 2 as query detects the bovine isoform 2 with high sequence similarity and a multiple alignment reveals the conservation of the sequence features covering the modified sites (Figure 3B). Such cross-specifies comparisons can provide the basis for the generation of new testable hypotheses, such as explaining the similarities or differences in protein function between different taxa due to the presence or absence of a given modification sites.

#### PRO and pathway analysis

**Figure 4:**
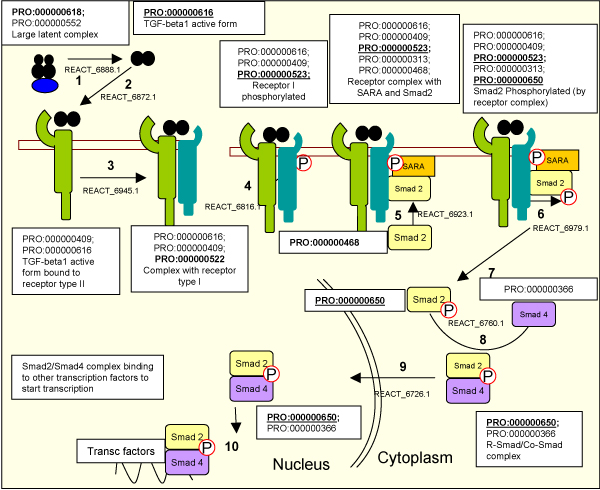
**PRO mapping to the Reactome TGF-beta signaling pathway**. The pathway described herein corresponds to REACT_6844. Each step in the pathway is described by a Reactome event ID. Bold PRO IDs indicate objects that undergo some modification that is relevant for function (the modified form is underlined). The names in the boxes represent the names of the complexes or molecules involved in the processes, not of the PRO terms.

The PRO curation of the TGF-beta signaling pathway illustrates the application of protein ontology in the context of pathway analysis. The states of a molecule are natural components of pathway ontologies or databases such as INOH Event Ontology [14] or Reactome [15]. As biomedical data expand, it will be increasingly important to explicitly represent these protein forms so that representations of attributes can be attached to the appropriate entities. Figure 4 shows the mapping of PRO terms to the associated Reactome events in the TGF-signaling pathway. The mapping of the entities involved in the pathway gives a more accurate and complete framework for researchers to analyze their data.

#### PRO and disease modelling

The PRO-pathway mapping supports modeling of the specific objects involved in a given disease in the pathway context. For example, it is possible to model cardiac myocyte apoptosis, a process in which activated caspase-3 generates the cleaved form of rho-associated protein kinase 1 (PRO:000000563). This form is constitutively active and promotes apoptotic signals, as has been observed both in the mouse myopathy model and in human heart failure patients [16,17]. Another example of disease modeling is the ability to link different protein variants that are associated to a common disease. Juvenile polyposis is an autosomal dominant syndrome predisposing to colorectal and gastric cancer. This syndrome is caused by variants of smad4 or BMP receptor type-1A [18]. Although smad4 is common to all TGF beta-related signaling pathways, BMP receptor type-1A is only active in the BMP-signaling sub-pathway; therefore, this information could potentially provide a clue for the specific pathway as well as the molecular mechanism that leads to the observed phenotype.

#### PRO and GO Complex

The GO Cellular Component includes protein complexes, which are defined in many cases on the basis of their component proteins. GO terms are species-neutral, as are the protein classes in PRO. PRO provides a means for creating the corresponding logical definitions in the GO complex ontology by using the precise PRO term that describes the form (modified or isoform) that occurs in the complex. Therefore, a GO complex can be defined as X complex *has_part *PRO1, *has_part *PRO2....*has_part *PRO*n*.

#### PRO and Cell Ontology

PRO also provides protein terms to Cell Ontology. Masci and collaborators have recently proposed to define some dentritic cell types (DC-CL) in terms of the proteins and protein complexes expressed on the cell surface by relating terms in DC-CL to terms for proteins in the PRO and to terms for protein complexes in GO [19].

### PRO dissemination and statistics

The PRO ontology file (in OBO format) and the PAF annotation file (in tab-delimited format) are disseminated through the PRO website [20], the OBO Foundry [21] and the National Center for Biomedical Ontology (NCBO) BioPortal [22]. PRO can be also downloaded in formats other than OBO such as OWL from the OBO Foundry [21]. Table 2 summarizes the release statistics with the total numbers of protein and PRO terms, which cover the TGF-beta signaling-related proteins (release 1.0), additional voltage-gated channel proteins as part of an ongoing collaboration with the Neuroscience Information Framework project [23] (release 2.0), and additional immunology-related proteins as part of the collaboration with the DC-CL project (current release 3.0). In the current PAF file, there are about 2350 annotations, covering 499 GO terms, and 1860 PMIDs. Table 3 provides more detailed statistics pertaining to the annotation of GO, PSI-MOD and SO terms.

**Table 2:** Statistics on Protein Ontology releases. PRO ontology coverage of proteins, and ProEvo and ProForm terms

**Number of**	**Pro.obo Releases**		
	
	1.0	2.0	3.0
Proteins	79	149	289
PRO terms	667	1023	1269
ProEvo terms	119	223	397
ProForm terms	548	800	872

**Table 3:** PRO annotation coverage by GO terms in release 3.0

**Ontology**	**OBO Relation**	# Terms*	**Example**	
GO Molecular Function	has_function	295	PRO:000000650 smad 5 isoform 1 phosphorylated 1	GO:0046332 SMAD binding
	NOT has_function	55	PRO:000000478 smad 5 isoform 2	GO:0046332 SMAD binding
GO Cellular Component Complex	part_of	62	PRO:000000178 RING-box protein 2 isoform 1	GO:0000151 ubiquitin ligase complex
	NOT part_of	5	PRO:000000179 RING-box protein 2 isoform 2	GO:0000151 ubiquitin ligase complex
GO Cellular Component	located_in	240	PRO:000000457 noggin isoform 1 cleaved 1	GO:0005615 extracellular space
GO Biological Process	participates_in	274	PRO:000000086 chordin isoform 1	GO:0001501 skeletal development
PSI-MOD	has_modification	172	PRO:000000650 smad 2 isoform 1 phosphorylated 1	MOD:00046 O-phospho- L-serine
SO	has_agent	305	TGF-beta receptor type-1 sequence variant 11	SO:1000097 mutation causing amino acid deletion

* Number of PRO terms under the umbrella of the corresponding ontology.

### Ongoing developments

One of the aims of the PRO consortium is to engage the biological community in the curation of the ontology and annotation. Thus, it is key to have a web-based editor that fulfils the requirement of a single interface for the curation of the ontology and annotation and that provides links to external resources and tools that will facilitate the curation task. A web-based ontology and annotation editor is under development and has been initially tested for community annotation during an annotation jamboree held in November, 2008, along with the PRO annual meeting. Based on the feedback, we are now enhancing the features and functionality of the editor. To maximize ontology usage, a web-based search interface will be developed that retrieves information from both the ontology and the annotation. This search interface will permit searching of the ontology and the annotation, plus other external links. Special functions will include: finding the equivalent isoforms (ortho-isoforms) in human and mouse, and the accompanying annotation; searching for the modified forms of a given protein that are non functional (with NOT *has_function *annotation); and searching for protein classes sharing a given domain.

## Conclusion

We have illustrated key aspects of the PRO framework through reference to proteins involved in the TGF-beta signaling pathway. The significance of the Protein Ontology is multi-fold: (1) ProEvo provides a structure to support formal, computer-based inferences based on shared attributes among homologous proteins; (2) ProForm helps to delineate the multiple protein forms of a gene; (3) PRO provides critical interconnections between existing OBO Foundry ontologies; (4) PRO can be integrated with or cross-referenced by other ontologies and/or databases, as for example, to better define objects in pathways or complexes or in disease modeling; and (5) the PRO framework allows the community to annotate proteins of interest. In summary, PRO offers a comprehensive picture of the protein realm by connecting protein evolution, function, modification, variants and disease. Finally, PRO can be adopted where data integration at the molecular level of proteins is needed, such as in systems biology or translational medicine.

## Methods

### Automated generation of PRO nodes

An automated process has been developed to generate preliminary PRO nodes (PrePRO) from PIR databases (PIRSF and iProClass [24]), UniProtKB [25], PANTHER [9] and MGI [26]. The automated process includes the following steps:

1. Retrieve all human and mouse proteins from UniProtKB.

2. Obtain splice and genetic variant nodes from the UniProtKB Features field.

3. Obtain orthologous relationship between mouse and human genes from MGI sources.

4. Obtain orthologous relationship between isoforms of mouse and human orthologous genes based on all-against-all BLAST alignment results.

5. Group orthologous genes according to PANTHER and/or PIRSF if applicable.

6. Extract PTMs and GO information from iProClass and literature sections of UniProtKB.

7. Arrange obtained information according to PRO framework.

Due to the dynamic nature of these data sources, we record the versioning of the sources to ensure accurate relationships of the mapping during PRO curation. PrePRO can be generated in both batch mode and interactively for a list of proteins to capture up-to-date information in the data sources. Each node is assigned a unique PrePRO ID. After curation a stable PRO ID is generated. The computationally-generated file is in OBO format, and OBO Edit 1.1 [27] has been used as the curation platform.

### Manual curation of PRO terms

Manual curation is needed because the PrePRO terms represent the protein entities and relationships as they are presented in the databases rather than representing the actual protein entities and their relationships. Curation involves (i) merging of nodes, for example where PIRSF and PANTHER families represent the same homeomorphic protein class, and (ii) creation of the protein forms after reviewing the literature and performing sequence analysis, for example to analyze what combination of modifications occurs in a specific form, and to determine what forms are equivalent in mouse and human (in the future potentially also in other organisms). In ProForm, the translation products originating from different mature transcripts of a gene or from the use of alternative initiation translation sites are referred to as isoforms. Equivalent isoforms in different organisms are collapsed into a single node. This assignment is based primarily on evidence in the literature and on protein sequence analysis. Genetic variants, including deletions/insertions and single nucleotide polymorphisms, are referred by PRO to as sequence variants. Isoforms are created based on the alternative product section of the UniProtKB entry, and variants (only for human entries) are created from the Seq_var field information. Only variants related to disease are included in the current version of the PRO. The final curated ProForm terms are based on experimentally observed entities, i.e., a type is added to the ontology only when there is at least one characterized sub-type.

### Curation of PRO names

All PRO term names are lower case except for certain standard abbreviations such as DNA, ATP, and GTP. These names are based on naming guidelines provided by nomenclature committees, literature, and UniProtKB [28], and the OBO Foundry [29], among others. The name used for a ProForm term is based on its parent node (see examples in Figure 2A).

### PRO definitions

All PRO terms have a definition that conforms to OBO foundry standards (see examples in Figure 2B). Whenever possible, the ProEvo definitions on the family level follow the form "A *parent term *with a core domain composition consisting of *domain 1, domain 2....*" with reference to conserved motifs and domain regions; in many cases examples are supplied. For example, PRO:000000026 smad anchor for receptor activation is defined as "A protein with a core domain composition consisting of a FYVE domain and a smad-binding domain that is involved in recruiting and presenting receptor-regulated smads to the receptor complex." [30]

Each PRO definition has source attribution to PubMed ID, PRO curator initials, or other resource ID. At the gene product level, the definition follows the format "A *parent term *that is a translation product of the *GENE NAME *gene." For example, PRO:000000125 sara is defined as: "A smad anchor for receptor activation that is a translation product of the ZFYVE9 gene." [PRO:CNA]

In many cases additional information is added. The reviewed section of UniProtKB contains curated gene names, providing standardization at least within certain taxon groups (e.g., within mammals, plants, fungi, prokaryotes). Within mammals, the human gene name is the reference for the gene name in PRO definitions, and other alternative names are added as synonyms. The definitions of isoforms follow the form "A *parent name *that is a translation product of a mature transcript of the *GENE NAME *gene. This form is represented by the *taxon *sequence UniProtKB:*AC-#*.", where "mature transcript" is a term defined by the Sequence Ontology (SO:0000233). Whenever possible, additional information about the presence or absence of domains or motifs is included as part of the definition. For example PRO:000000315 sara isoform 3 is defined as: "A sara that is a translation product of the mature transcript of ZFYVE9 gene, and lacks the smad-binding domain. This form is represented by the human sequence UniProtKB:O95405-3." [PRO:CNA]

### PRO annotations

The annotation to the PRO terms was done according to PAF formatting guidelines [31] and the GO Annotation Guide [32].

## Competing interests

The authors declare that they have no competing interests.

## Authors' contributions

CNA carried out curation of PRO, coordinated the curation team, participated in the design of the study and drafted the manuscript. HL developed the script to generate the automatic PRO file (prePRO) and drafted the details of this procedure in this manuscript. DAN provided methods to compare isoforms and provided quality assurance for the ontology terms used in PRO. WCB provided critical insight about ProEvo and is a curator of ProEvo terms. HJD provided GO annotation for mouse proteins and helped in the design of the PRO association file. JAB participated in the design of the study. BS advised on the ontology terms and relations, and helped in the draft of the manuscript. CHW coordinated the PRO consortium activities, and participated in the design of the study. All authors read, critically revised and approved the final manuscript.
